# Early Inflammatory Status Related to Pediatric Obesity

**DOI:** 10.3389/fped.2019.00241

**Published:** 2019-06-18

**Authors:** Cristina Oana Mărginean, Lorena Elena Meliţ, Dana Valentina Ghiga, Maria Oana Mărginean

**Affiliations:** ^1^Department of Pediatrics, University of Medicine, Pharmacy, Sciences, and Technology, Târgu Mureş, Romania; ^2^Department of Medical Informatics and Biostatistics, University of Medicine, Pharmacy, Sciences and Technology, Târgu Mureş, Romania; ^3^Department of Pediatric Cardiology, University of Medicine, Pharmacy, Sciences and Technology, Târgu Mureş, Romania

**Keywords:** children, overweight, obese, neutrophil to lymphocyte ratio (NLR), platelet to lymphocyte ration (PLR)

## Abstract

**Background:** Obese individuals are often in a chronic inflammatory condition due to the malfunction of immune-related activities in the adipose tissue, involving a transient infiltration of neutrophils within the abdominal fat and their binding to adipocytes. Neutrophil to lymphocyte ratio (NLR) and platelet to lymphocyte ratio (PLR) are considered cost-effective markers for the detection of subclinical inflammation. Our study intends to assess the early stages of inflammation associated with overweight and obesity in children.

**Materials and Methods:** We performed a prospective study with 164 children, aged between 5 and 18 years, admitted to a Pediatric Tertiary Hospital in Romania between January 2018 and January 2019. The patients were divided according to body mass index (BMI) into two groups: Group 1: 77 overweight and obese children (BMI percentile ≥85), and Group 2: 87 children with a normal BMI, in order to evaluate the correlation between BMI and laboratory parameters (CBC, ESR, transaminase, total protein, albumin, and blood glucose levels), inflammatory biomarkers, NLR and PLR, and changes in abdominal ultrasound findings.

**Results:** We found that the leukocyte, lymphocyte, erythrocyte, platelet, CRP, and transaminase levels were significantly higher in the overweight/obese group (*p* = 0.0379, *p* = 0.0002, *p* = 0.0003, *p* = 0.0006, *p* < 0.0001, *p* = 0.0332, and *p* < 0.0001, respectively). No significant statistical differences between the two groups in terms of neutrophil, hemoglobin, albumin, total protein, and glycemia levels were noted (*p* > 0.05). Moreover, NLR and PLR did not differ significantly between the two groups (*p* = 0.4674 and *p* = 0.9973, respectively).

**Conclusions:** Obesity is associated with systemic low-grade inflammation which is reaching alarming rates worldwide among both children and adults. Our study proved that leukocyte, lymphocyte, erythrocyte, and platelet levels are significantly higher in overweight/obese children, emphasizing the inflammatory status related to this condition. Therefore, obesity-related studies involving pediatric patients are of major interest in order to develop appropriate methods to prevent the development of further complications in adulthood.

## Introduction

Obesity is a current global health problem in both children and adults and causes a significant burden. Most studies investigating obesity involves adults; however, it is essential for studies to focus on childhood obesity in order to prevent its associated complications and further development in adulthood. The incidence of this nutritional disorder in children has reached alarming rates worldwide. In Romania, one in four children were found to be either overweight or obese (1). Its etiology is complex, involving an interaction between genetic susceptibility and environmental or “obesogenic” factors, which play a key role in triggering obesity, representing therefore the basis for successful interventions (2).

It is well-documented that obese individuals express a chronic inflammatory status; obesity-related complications, such as cardiovascular disease, type 2 diabetes mellitus, metabolic syndrome, and non-alcoholic steatohepatitis were proven to be results of obesity-associated low-grade inflammation (3, 4). This obesity-related inflammation is due to the malfunction of immune-related activities in the adipose tissue, involving a transient infiltration of neutrophils within the abdominal fat and their binding to adipocytes (5). This process may precede macrophage infiltration similar to that in other inflammatory conditions (5, 6). Moreover, neutrophils are the most important and abundant subtype of white blood cells in human peripheral blood. Neutrophils and chronic inflammation seem to be linked to chronic hypertension and obesity (7), with the total count of circulating neutrophils being increased in obese individuals (8). Besides neutrophils, leukocytes are also associated with obesity-induced chronic inflammatory status, being equally involved in related comorbidity development. Based on this premise, obese individuals without comorbidities are considered to represent a special subgroup in the early stage of inflammation similar to overweight individuals (9).

Multiple serum markers are said to be associated with low-grade chronic inflammation. Neutrophil to lymphocyte ratio (NLR) is a recently discovered, cost-effective marker for the detection of subclinical inflammation that correlates with C-reactive protein (CRP) levels (10–12). This useful marker has been related to multiple inflammatory conditions, cardiovascular diseases, and cancer (13, 14). NLR was shown to be directly related to the degree of inflammation (15). Platelet to lymphocyte ration (PLR) is another biomarker that can be calculated based on the complete blood count (CBC) and has been proven to be useful in the diagnosis and monitoring of several systemic inflammatory processes (16–19). PLR is an indicator of the balance between inflammation and thrombosis. Thus, the inflammatory status results in accelerated megakaryocyte proliferation and associated thrombocytosis. Moreover, increased platelet counts and decreased lymphocyte counts have been shown to be related to both aggregation and inflammation, and thus, represent risk indicators (20, 21). Other blood parameters, such as lymphocytes, monocytes, hemoglobin, erythrocyte sedimentation rate (ESR), total proteins, albumin, iron, cholesterol, triglycerides, and transaminases, and different gene polymorphisms, are related to childhood obesity and overweight (22–29).

***The aim*** of this study was to assess different blood parameters associated with the low grade inflammatory status in overweight and obese children in order to detect the early stages of inflammation related to this nutritional disorder.

## Materials and Methods

### Study Sample

We performed a prospective study with 191 pediatric patients aged between 5 and 18 years, referred to a Pediatric Tertiary Hospital in Romania, between January 2018 and January 2019 that were assessed on a 1 day admission chart system, without requiring long time hospitalization. However, the parents of only 173 children agreed to sign the informed consent form for their children to be included in the study. After selection according to age and sex, only 164 children were finally included in the study. The children were divided according the value of body mass index (BMI) into two groups: group 1, the study group: 77 overweight and obese children (overweight children: BMI percentile ≥85 and <95, and obese children: BMI percentile >95), and group 2, the control group: 87 children with normal BMI (percentile ≥5 and <85). The inclusion criteria for both groups was an age of between 5 and 18 years. In the study group we included children with a BMI percentile >85, while in the control group we included those with a BMI percentile ≥5 and <85 (30–32). The exclusion criteria consisted of age below 5 years, secondary obesity, patients with obesity-related complications, chronic disorders, infectious diseases, incomplete data, and patients whose parents refused to sign the informed consent form.

All patients underwent a thorough anamnesis and clinical exam; blood parameters were also measured: CBC, ESR, CRP, transaminase, total protein, albumin, and blood glucose levels. Inflammatory biomarkers, such as NLR and PLR, were calculated by dividing the neutrophil count and platelet count, respectively, by the lymphocyte count. The abdominal ultrasound was performed in all children by a single trained clinician. The laboratory parameters were assessed using a Cobas Integra 400 plus automated analyzer, Roche Diagnostics GmbH, Mannheim, Germany. The ultrasound exams were performed with a probe, with variable frequencies between 2.5 and 6 MHz, and an S8 General Electric Machine.

All parents/caregivers signed the informed consent for their children. The study was approved by the Ethics Committee of the University of Medicine and Pharmacy of Târgu Mureş (No 329/November 17th 2017), and it was performed according to the principles of the Helsinki Declaration.

### Statistical Analysis

The children' characteristics are presented as means ± standard deviation and medians.

The statistical analysis comprised of descriptive statistical analysis (frequency, media, median, and standard deviations) and inferential statistical elements. D'Agostino & Pearson's tests were applied in order to identify the distribution of the series of analyzed data. We applied the Student *t*-test for unpaired data, and the Mann-Whitney test for comparison of the medians. A Chi squared test was used for association determination. The significance threshold was considered at a *p*-value of 0.05. The statistical analysis was performed using GraphPad Prism 7 free trial version, GraphPad Software Inc., California, USA.

## Results

Among the 164 children included in the study, the mean age for the study group was 10.79 ± 3.503 years, whereas that for the control group was 12.33 ± 3.516 years (*p* = 0.0052). Regarding residence area, we found the same distribution of obese children among rural and urban areas (*p* = 0.8338), without a significance influence of the social status in the development of obesity. According to sex distribution, being overweight/obese was more common in boys (*p* = 0.0083). Thus, our sample is age- and sex-matched. We also assessed the birth weight, identifying a higher value for control group, 3.278 ± 1.070 kg in comparison to 3.310 ± 0.5603 kg for obese children, but without statistical significance (*p* = 0.1400). The current weight and BMI of the children included in our study were significantly higher for obese group (*p* < 0.0001).Thus, the mean value of the current weight was 57.78 ± 20.94 kg for obese children versus 45.36 ± 14.46 kg for control group, while the mean value of BMI in case of obese children was of 25.82 ± 4.518 kg/m^2^ compared to 18.92 ± 2.934 kg/m^2^ for normal weight children. Contrariwise, the height was higher for control group, though without statistical significance (*p* = 0.0263), with a mean value of 152.7 ± 16.19 cm for control group in comparison to 146.8 ± 17.83 cm for obese children. All these descriptive parameters along with the values of the laboratory parameters are mentioned in Table 1.

**Table 1:** The descriptive analysis of the blood parameters in the two groups.

**Laboratory parameters**	**Study group(*n* = 77) Mean ± SD**	**Control group (*n* = 87) Mean ± SD**	***p*-value**
Age (years)	10.79 ± 3.503	12.33 ± 3.516	^*^0.0052
Birth weight (kg)	3.310 ± 0.5603	3.278 ± 1.070	^*^0.1400
Current weight (kg)	57.78 ± 20.94	45.36 ± 14.46	**<0.0001**
Height (cm)	146.8 ± 17.83	152.7 ± 16.19	0.0263
BMI (kg/m^2^)	25.82 ± 4.518	18.92 ± 2.934	**<0.0001**
Leukocytes (10^3^/μl)	7.932 ± 2.885	7.162 ± 1.896	^*^0.0379
Neutrophils (10^3^/μl)	3.848 ± 2293	3.309 ± 2210	^*^0.1063
Lymphocytes (10^3^/μl)	2871 ± 1042	2382 ± 743.3	^*^0.0002
Hemoglobin (g/dl)	13.55 ± 1.277	13.45 ± 1.193	^*^0.1776
Erythrocytes (10^6^/L)	4.989 ± 0.3986	4.811 ± 0.3863	^*^0.0003
Platelets (10^3^/μl)	338.0 ± 100.3	290.0 ± 66.27	**0.0006**
Albumin (g/dL)	4.892 ± 0.3255	4.831 ± 0.3290	0.2295
Total proteins (g/dL)	7.511 ± 0.4958	7.470 ± 0.4247	0.5676
AST (UI)	26.87 ± 23.78	22.37 ± 12.18	^*^0.0332
ALT (UI)	27.17 ± 43.14	14.68 ± 9.655	^*^<0.0001
Glycaemia (mg/dl)	86.33 ± 9.527	84.87 ± 9.171	^*^0.3338
NLR	1.577 ± 0.8019	1.878 ± 1.527	^*^0.4673
PLR	125.9 ± 44.77	131.5 ± 47.21	^*^0.9973
ESR	12.55 ± 7.965	11.32 ± 8.690	^*^0.2122
CRP (mg/l)	4.508 ± 2.839	1.938 ± 4.729	^*^<0.0001

*ALT, alanine aminotransferase; AST, aspartate aminotransferase; BMI, body mass index; CRP, C-reactive protein; NLR, neutrophil/ lymphocyte ratio; ESR, erythrocyte sedimentation rate; n, number; PLR, platelets/lymphocyte ratio; SD, standard deviation*;

* *Mann-Whitney test was used. n = number. Bold represents the statistically significant values*.

**Figure 1:**
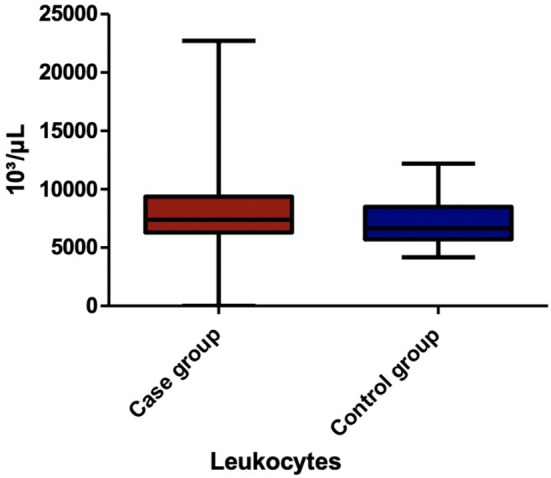
The comparison of leukocyte count between the two groups.

**Figure 2:**
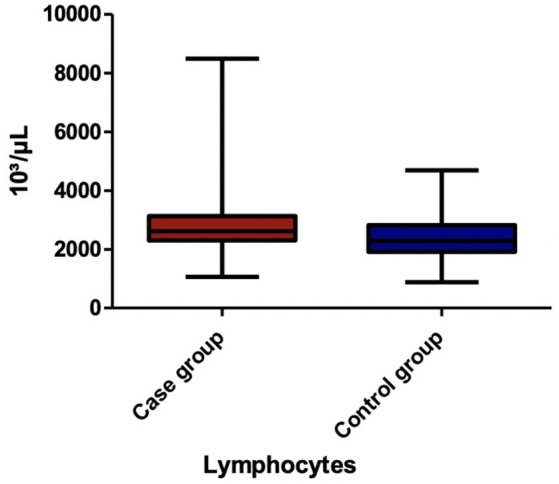
The comparison of lymphocyte count between the two groups.

**Figure 3:**
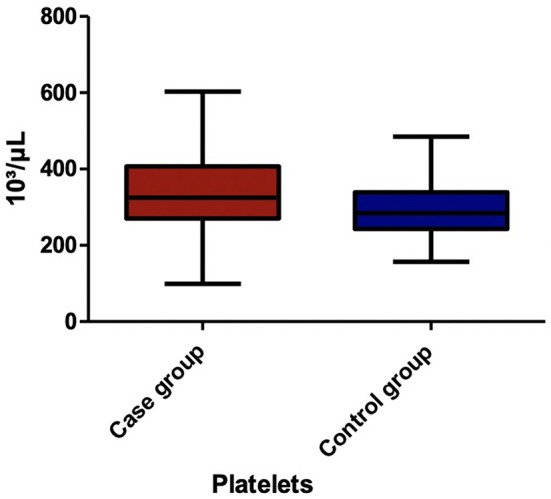
The comparison of platelet count between the two groups.

**Figure 4:**
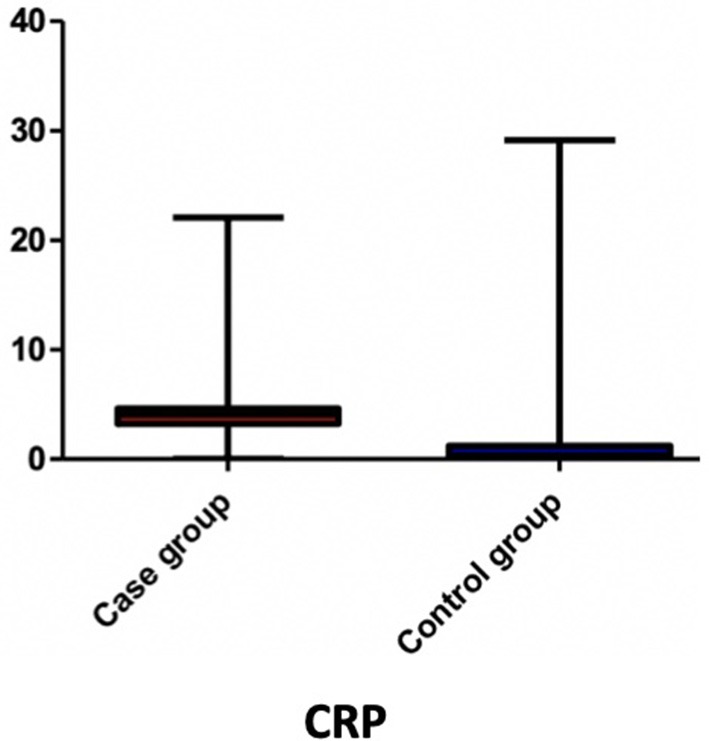
The comparison of CRP value between the two groups.

Among the blood parameters, we found that leukocyte (Figure 1), lymphocyte (Figure 2), erythrocyte, platelet (Figure 3), CRP (Figure 4), and transaminase levels (AST and ALT) were significantly higher in the overweight/obese children in contrast to the control group (*p* = 0.0379, *p* = 0.0002, *p* = 0.0003, *p* = 0.0006, *p* < 0.0001, *p* = 0.0332, and *p* < 0.0001, respectively).

Nevertheless, we did not encounter any significant statistical differences between the two groups in terms of neutrophil, hemoglobin, albumin, total protein, erythrocyte sedimentation rate (ESR), and glycemia levels (*p* = 0.1063, *p* = 0.1776, *p* = 0.2295, *p* = 0.5676, *p* = 0.2122, and *p* = 0.3338, respectively). Moreover, NLR and PLR did not differ significantly between the two groups (*p* = 0.4674 and *p* = 0.9973, respectively).

Regarding the results of the abdominal ultrasound, we found pathological changes, such as hepatomegaly and hepatic steatosis, in 77.92% of the overweight/obese children, whereas, in the control group, only three children were identified to have hepatomegaly (3.44%). Thus, hepatomegaly and hepatic steatosis were significantly more frequent in the overweight/obese group than in the normal BMI group (*p* > 0.0001) (Table 2).

**Table 2:** Comparative analysis of liver ultrasound findings between the two groups.

**Abdominal ultrasound**	**Study group (77 cases)**	**Control group (87 cases)**
Hepatomegaly	13	3
Normal	17	84
Steatosis	19	0
Hepatomegaly, steatosis	27	0
Steatohepatitis areas surrounding the gallbladder	1	0

## Discussions

The World Health Organization stated that the prevalence of obesity worldwide has nearly tripled since 1975 (33). Thus, in 2016, 41 million children below the age of 5 years were overweight or obese (33). The prevalence of obesity increases with age, with over 340 million children and adolescents aged between 5 and 19 years being affected with this condition in 2016 (33). Obesity is an important leading cause of mortality worldwide, being associated with increased risk for heart failure, stroke, skeletal system diseases, and malignancies (33).

The adipose tissue has been proven to contribute to both the initiation and maintenance of systemic inflammation (5, 34). Adipose tissue-related inflammation leads to a wide variety of immune responses, involving neutrophil participation in the early phases followed by macrophage involvement and mast cell polarization (35, 36). Studies performed on mice proved that the neutrophil count significantly increases within the adipose tissue even in the first days after the initiation of a high fat diet (37), suggesting that obesity, even in its early stages, is associated with systemic inflammation. Similar findings were also reported in humans in the study of Tam et al. (38), who underlined that acute lipid overload is related to the increase of inflammatory biomarkers, such as CRP and monocyte chemoattractant protein-1. Our study sustains the findings of Tam et al. regarding the CRP, which was significantly higher in the obese/overweight children included in this research. Recently, a wide range of biomarkers were found to be related to low-grade systemic inflammations, such as CBC parameters, NLR, PLR, mean platelet volume, and CRP among others. Thus, it was proven that the peripheral blood of otherwise healthy overweight/obese individuals exhibit characteristics of a low-grade inflammation (6).

White blood cells were shown to be associated with the development of metabolic syndrome (39, 40). A study performed with 6,700 patients found positive correlations between waist circumference and leukocyte, lymphocyte, neutrophil, and platelet levels, and medium platelet volume (41). Moreover, another study including 223 subjects showed that an increase in BMI results in increased leukocyte, lymphocyte, platelet, and neutrophil counts (42). Similar to the findings of the previously mentioned study, our study showed a significant correlation between being overweight/obese and leukocyte, platelet, lymphocyte, and erythrocyte counts. However, it failed to show a significant association of these conditions with the neutrophil count. Despite the fact that NLR and PLR are immune response markers related to chronic inflammation (19, 43), we did not find significant correlations between these markers and obesity/overweight. The lack of correlation between NLR and BMI in our study might be explained by the significant increase in lymphocyte count among overweight/obese children. Most likely, PLR did not correlate with an increased BMI in our study due to the significant increase in both platelet and lymphocyte counts in the overweight/obese group. Recent studies emphasize the role of NLR as a potential inflammation marker in not only cardiac and non-cardiac disorders, but also autoimmune conditions and infections (44, 45). Inflammatory status is reflected by neutrophil counts, while the lymphocyte count is linked to the nutritional status and general stress (46). The increase in neutrophil count was proven to be directly related to the degree of obesity (47). Thus, the fact that in our study we did not find a significant increase in neutrophil count among the study group might be explained by the young age of our subjects, resulting in an insufficient amount of time for chronic inflammation to occur. Moreover, multiple studies have focused on assessing the role of NLR and PLR in patients with related-comorbidities; a study performed with 155 obese and non-obese patients with obstructive sleep apnea found significantly higher values in terms of NLR among obese individuals (46). Metabolic syndrome related obesity is another disorder commonly associated with chronic inflammation that was found to be related with NLR (48). Nevertheless, the findings remain contradictory because similar to our study, Bahadir et al. also failed to prove an association between NLR and obesity or metabolic syndrome (49). Diabetes mellitus and its related metabolic malfunctions are also associated with an increased NLR (50).

Most obesity-related studies have been performed using adult participants and stated that the wide range of associated comorbidities hinder the elucidation of a cause-effect link between inflammation and obesity (51). Similar to our study, Aydin et al. performed a study with 187 children (130 obese individuals and 57 healthy controls) (51). They found that the age of obese children was lower than that of the healthy controls in their study. Regarding blood parameters, the authors showed that lymphocyte and neutrophil counts, and NLR were significantly higher in obese children. Similarly, our study also showed a significant increase in lymphocyte count among overweight/obese children, but it failed to show significant differences in terms of neutrophil count and NLR between the normal BMI children and those with high BMI. Moreover, the previously mentioned study (51) did not identify any significant differences between obese and normal weight children with respect to PLR, as in our study. These contradictory findings might be explained by the fact that pediatric cases are rarely accompanied by obesity-related complications.

The limitations of our study consist of the relatively small sample size and the lack of correlation with dietary habits and the assessment of related complications. Conversely, this study will make a significant contribution to the existing literature as it involved pediatric patients and was able to identify the risk factors associated with the early phases of obesity-related inflammatory process. Moreover, to the best of our knowledge, this is the first study in Romania that assessed the role of CBC parameters, NLR, and PLR in overweight/obese children.

## Conclusions

Obesity is associated with systemic low-grade inflammation and is reaching alarming rates worldwide among both children and adults. Our study proved that leukocyte, lymphocyte, erythrocyte, and platelet counts are significantly higher in overweight/obese children, emphasizing the early inflammatory status related to this condition. Therefore, obesity-related studies involving pediatric patients are crucial in order to develop appropriate methods for preventing the development of further complications in adulthood.

## Ethics Statement

The study was approved by the Ethics Committee of the University of Medicine and Pharmacy of Târgu Mureş (No 329/ November 17th 2017), and it was performed according to the principles of the Helsinki Declaration. The consent procedure used in our study consisted in an informed written consent that was signed by all parents/caregivers for their children prior to the inclusion in the study. The study was also explained to the children signed and we obtained their verbal assent.

## Author Contributions

CM, LM, and MM conceptualized and designed the study, drafted the initial manuscript, and reviewed, and revised the manuscript. MM and LM designed the data collection instruments, collected data, carried out the initial analyses, and reviewed and revised the manuscript. DG performed the statistical analysis. All authors approved the final manuscript as submitted and agree to be accountable for all aspects of the work.

### Conflict of Interest Statement

The authors declare that the research was conducted in the absence of any commercial or financial relationships that could be construed as a potential conflict of interest.

## Abbreviations

ALT: alanine aminotransferase
AST: aspartate aminotransferase
BMI: body mass index
CBC: complete blood cellular
CRP: C-reactive protein
ESR: erythrocyte sedimentation rate
n: number
NLR: neutrophil/lymphocyte ratio
PLR: platelets/lymphocyte ratio
SD: standard deviation.
